# Hypospadias Repair: A Single Centre Experience

**DOI:** 10.1155/2014/453039

**Published:** 2014-01-20

**Authors:** Mansoor Khan, Abdul Majeed, Waqas Hayat, Hidayat Ullah, Shazia Naz, Syed Asif Shah, Tahmeedullah Tahmeed, Kanwal Yousaf, Muhammad Tahir

**Affiliations:** Plastic & Reconstructive Surgery, Hayatabad Medical Complex, IV Hayatabad, P.O. Box 25100, Peshawar, Pakistan

## Abstract

*Objectives*. To determine the demographics and analyze the management and factors influencing the postoperative complications of hypospadias repair. *Settings*. Hayatabad Medical Complex Peshawar, Pakistan, from January 2007 to December 2011. *Material and Methods*. All male patients presenting with hypospadias irrespective of their ages were included in the study. The data were acquired from the hospital's database and analyzed with Statistical Package for Social Sciences (SPSS). *Results*. A total of 428 patients with mean age of 8.12 ± 5.04 SD presented for hypospadias repair. Midpenile hypospadias were the most common. Chordee, meatal abnormalities, cryptorchidism, and inguinal hernias were observed in 74.3%, 9.6%, 2.8%, and 2.1% cases, respectively. Two-stage (Bracka) and TIP (tubularized incised urethral plate) repairs were performed in 76.2% and 20.8% of cases, respectively. The most common complications were edema and urethrocutaneous fistula (UCF). The complications were significantly lower in the hands of specialists than residents (*P*-value = 0.0086). The two-stage hypospadias repair resulted in higher complications frequency than single-stage repair (*P* value = 0.0001). *Conclusion*. Hypospadias surgery has a long learning curve because it requires a great deal of temperament, surgical skill and acquaintance with magnifications. Single-stage repair should be encouraged wherever applicable due to its lower postoperative complications.

## 1. Introduction

Hypospadias is the most common congenital abnormality of the urethra affecting 1 : 300 live male births worldwide. The incidence is on the rise with the increasing environmental pollution as the suspected cause [1]. In 1993, the Birth Defects Monitoring Program (BDMP) has reported a doubling of the rates of hypospadias since 1970s in the United States [2]. Hypospadias is the abnormal location of the urethra on the ventral surface of the penis with variable association with the aborted development of the urethral spongiosum, ventral prepuce, and penile chordee [3]. By meatal location hypospadias is classified as anterior (glanular and subcoronal), mid-penile (distal penile, midshaft, and proximal penile), and posterior (penoscrotal, scrotal, and perineal) accounting for 50%, 30%, and 20%, respectively [4]. Those cases in which multiple procedures are performed with suboptimal results are termed “crippled cases” [5]. One fourth of the hypospadias are associated with chordee [6]. Devine and Horton classified chordee into type I (skin tethering), type II (fibrotic dartos and buck's fascia), type III (corporal disproportion), and type IV (congenital short urethra) [7, 8].

In the management of hypospadias preoperative assessment is of prime importance which should include measurement of the size of the phallus, glans cleft (flat, incomplete, or complete), location and size of the meatus (type of hypospadias and meatal stenosis or mega-meatus), urethral plate width (<1 cm or ≥1 cm), type of chordee, prepuce (complete, incomplete, circumcised), penile torsion (clockwise, anticlockwise), shape of the scrotum (normal, penoscrotal transposition), and associated anomalies (cryptorchidism, inguinal hernia, persistent Mullerian structures) [4, 9]. Hypospadias can be associated with other urogenital tract anomalies such as pelviureteric junction (PUJ) obstruction, vesicoureteric reflux and renal agenesis which should be excluded by ultrasonographic scan in every hypospadias patient [4, 10, 11]. Proximal hypospadias with cryptorchidism, enlarged utricle, or penile size <2.5 cm should be investigated for intersex disorders by ultrasonography, hormonal profile, and karyotyping [12].

Timing of surgery is decided on the basis of anaesthesia's risk, penile size, and psychosocial development of the infant. The tolerance to anaesthesia is good at the age of 6 months. The difference in the penile length at one year and preschool age is 8 millimetre only. After 18 months the children enter a behavioural phase of development uncooperative for hospitalization. In the background of these facts, using microsurgical instruments and magnification, 6–18 months is the most suitable age for hypospadias repair. If the surgery is not performed during this age then the next window of surgery is preschool age (3-4 years) when the child starts cooperating with treatment [4, 13].

Minimal tissue trauma, minimal/pin-point use of cautery, and well-vascularised tension free repair of all layers with epithelial inversion are the general principles of hypospadias repair. The goal of hypospadias repair is to build confidence in the child by creating a straight penis with a slit-like meatus at the tip of the glans and a urethra of uniform calibre and adequate length, reconstructing a symmetrical glans and penile shaft and achieving projectile stream and normal erection [4].

In patients with chordee the curvature is quantified peroperatively by Horton test after degloving the penis into mild (10°–20°), moderate (30°–40°), and severe (>50°). Most of the hypospadiologists do not address the mild chordee although they agree on correcting the moderate and severe forms through dorsal (plication) and ventral approach (excising the fibrous tissue), respectively [14, 15]. The chordee correction starts by penile degloving, and urethral plate and proximal urethral mobilization. The persistent chordee after these maneuvers is either due to urethral plate tethering or corporal disproportion. The former is corrected by urethral plate transaction while the later anomaly requires dorsal plication/Nesbit procedure [13]. The dorsal nerve branches from 11 and 1 o'clock positions to the 5 and 7 o'clock positions, making the 12 o'clock position ideal for dorsal plication during correction of the chordee [16]. In cases of hypoplastic penis where dorsal plication can unacceptably shorten the length then tunica release and dermal or tunica grafts can be considered [13].

The choice of the procedure is based on the characteristics of the urethral plate irrespective of the meatal location. The hypospadias repairs can be classified into single-stage procedures and two-stage urethral plate substitution procedure (Bracka's repair). The single-stage procedures are (a) urethral plate tubularization (glanular approximation and Snodgrass repair) and (b) urethral plate augmentation (onlay flap and Snodgraft repair) [13].

When the urethral plate does not need transaction then it can be tubularized either by Zaonz's GAP (glanular approximation procedure) when the plate is wide and deep or by Snodgrass's TIP in cases of narrow, shallow urethral plates with occasional bands. In cases of inelastic urethral plate where the midline releasing incision is not expected to widen the plate then substantial augmentation can be performed either by Duckett's onlay preputial island flap or a more popular Snodgraft repair by quilting a full thickness preputial skin graft in the dorsal defect after the releasing midline incision. The Snodgraft repair is also indicated in conical glans where the Snodgrass (TIP) midline incision is extended beyond the distal limit of the glans groove to achieve an apical meatus, inciting meatal stenosis unless grafted [13].

The urethral substitution procedures come into play when the chordee is corrected by urethral plate transaction. Due to the long term complications of Transverse Preputial Island Flap (Duckett's procedure) for the substitution of the whole circumference of the transacted urethra has fallen out of favour for the Bracka's two-stage repair where full thickness preputial skin graft is used [13].

Despite the controversial status, most of the hypospadiologists favour the urinary diversion and postoperative dressing. The penile block along with the general anaesthesia should be used to achieve successful postoperative analgesia.

Follow-up protocol after hypospadias repair is designed to institute a balance between its pros (early detection of complications) and cones (psychological concerns by repeatedly reminding with the patient the abnormality). After removal of the stent at one week postoperatively the patient is followed for 1, 3 and 6 months intervals and then yearly for two years. For the assessment of long term results patient can be followed up to midteen age [4, 13].

Biotechnology development in hypospadias surgery is directed toward the application of LASER shouldering (to replace conventional suturing techniques), robotics (for removal of human errors, e.g., hand tremors), tissue engineering (for urethral regeneration), and the use of tubular acellular collagen seeded with urothelial cells [17, 18].

Hypospadias surgery has a long learning curve. A reliable operator has the temperament for the hypospadias surgery, with an annual case load of at least 40–50, and has mastered six common techniques [4, 13]. This paper is aimed to present and analyze the demographics, protocols, techniques, complication of hypospadias repair, and its effect modifiers at our centre.

## 2. Material and Methods

After approval of the study protocol from the ethical committee, all male patients, irrespective of their age, managed for hypospadias from January 2007 to December 2011 at the Plastic and Reconstructive Surgery Department of Hayatabad Medical Complex Peshawar, Pakistan, were included in the study. Patients with comorbidities (coagulation disorders, diabetes mellitus, and disorders of sex development) and those patients who were lost in the followup were excluded from the study population to omit bias from the study results. All the demographic, clinical presentation, laboratory studies, surgical treatment, complications, and their management data were collected from the department's record sheets. To stratify the results, surgeons were divided into two groups; residents (trainees) and specialists (fellow plastic surgeons). Residents performed surgeries under direct or indirect supervision of the specialist. The data were organized, analyzed, and presented with the help of Statistical Package for Social Sciences (SPSS). The qualitative variables were presented as frequencies and percentages while quantitative variables were analyzed as means ± SD. All the important variables were stratified against age, type and severity of hypospadias, duration of surgery, type of surgical procedure, and experience of the surgeon to see the effect modifiers. The results were projected as tables and figures.

## 3. Results

A total of 428 male patients consisting of 96.3% primary cases and 3.7% secondary cases fulfilled the inclusion criteria. Patients with age ranging from 1 to 40 years with mean age of 8.12 ± 5.04 SD presented for hypospadias repair. Thirty-seven (8.6%) patients had the positive family history for hypospadias. Increasing trend of hypospadias patients presenting for surgery was observed during the study period with the highest of 103 (24.1%) patients presented in 2011. Only 1.87% and 34.6% patients were operated in 1-2 years, 3–5-year age windows while the rest presented in ≥6 years of age.

**Figure 1:**
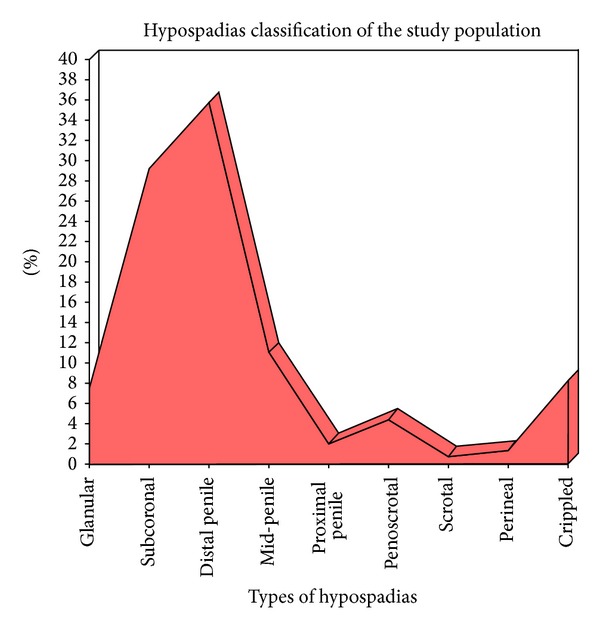
Hypospadias classification of the study population.

**Figure 2:**
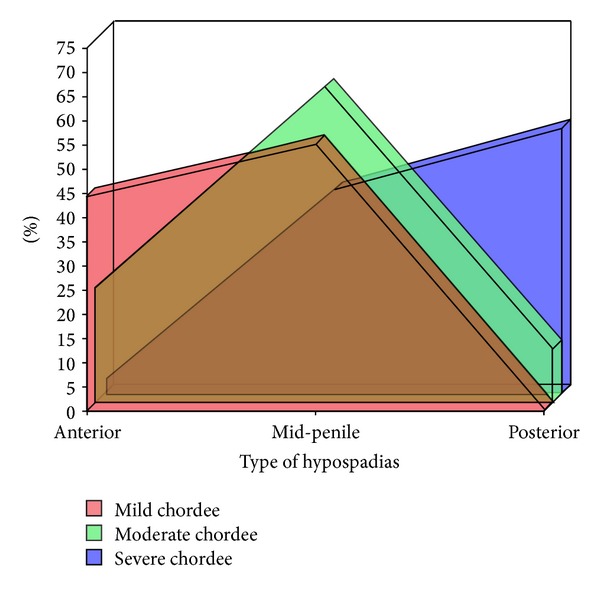
Type of hypospadias and degree of chordee.

Mid-penile hypospadias (distal penile, midshaft, and proximal penile) was the most common type in the study population accounting for 48.6% (*n* = 208) patients followed by anterior hypospadias (glanular and subcoronal) in 36.4% (*n* = 156) cases, which is consistent for all geographical locations of the study population (Figure 1). Of the total study population, 74.3% (*n* = 318) of the hypospadias was associated with some degree of chordee. The most common type of chordee was the mild degree (<20°) with a count of 51.4% (*n* = 220) cases. Severe chordee was found in 7.5% (*n* = 32) cases. The chordee was present in 100% cases of penoscrotal and perineal hypospadias and the least common for glanular hypospadias accounting for 54.8% cases (Figure 2). Meatal abnormalities associated with the hypospadias were observed in 9.6% (*n* = 41) patients. Meatal stenosis was the most common meatal abnormality affecting 9.1% (*n* = 39) patients while mega-meatus was observed in 0.5% (*n* = 2) cases. Perineal and glanular hypospadias were the most commonly associated with meatal stenosis in 16.7% and 16.1% cases, respectively. Cryptorchidism was observed in 2.8% (*n* = 12) cases. A total of 2.1% (*n* = 9) hypospadias were associated with inguinal hernias.

Of the total 428 cases, 76.2% (*n* = 326) patients' hypospadias were repaired in two stages (Bracka) while 20.8% (*n* = 89) were subjected to TIP repair. Two-stage hypospadias repair was the predominant surgical procedure for all types of the hypospadias. For 110 cases without chordee, 53.6% (*n* = 59) hypospadias were managed by different single-stage repairs with TIP the predominant procedure performed in 54 (49.1%) cases. Two-stage (Bracka) repair was performed in 46.4% (*n* = 51) in 110 hypospadias without chordee for narrow urethral plate and incomplete glans cleft.

Of the total 428 hypospadias, 66.1% (*n* = 283) were performed by specialist plastic surgeons while 33.9% (*n* = 145) were operated on by residents. Specialists performed two-stage (Bracka) and single-stage (i.e., TIP, MAGPI, and Mathieu's) repairs in 67.14% and 32.9% cases, respectively. While residents performed two-stage (Bracka) and TIP repair in 93.8% and 6.2% cases.

For 50.7% (*n* = 217) cases duration of surgical procedure was >60 minutes. Duration of surgical procedure for specialists was ≤60 minutes in 58.7% cases while for the residents it was >60 minutes in 68.7% cases. In 90% (*n* = 385) cases the hospital stay was 3 days.

The frequency of postoperative acute complications was edema (28.3%), bleeding (4.4%), surgical site infection 4.2%, wound dehiscence (4.2%), and partial graft (1.4%) loss, which were successfully managed conservatively (Table 1).

The most common chronic complication was UCF which was initially observed in 38.8% (*n* = 166), of which 50.6% (*n* = 84) and 15.1% (*n* = 25) were managed by single surgical procedure and multiple surgical procedures, respectively. While 31.33% (*n* = 52) closed spontaneously during one to three months postoperatively leaving the corrected frequency of UCF as 26.6%. The second most common chronic complication was meatal stenosis observed in 5.6% (*n* = 24) patients. The UCF frequency was the highest for proximal hypospadias (scrotal, penoscrotal and perineal) as projected in Table 2.

The overall complication frequencies for specialists and residents were 56.9% (*n* = 161) and 70.34% (*n* = 102) which is statistically very significant with a *P* value of 0.0086 with a confidence interval of 95%, calculated by Fisher's exact test (Table 3). The corrected fistula rates (excluding those which healed spontaneously) for specialists and residents were 23.32% (*n* = 66) and 33.10% (*n* = 48), respectively, which is statistically significant with a *P*-value of 0.0374, calculated by Fisher's exact test with a confidence interval of 95% (Table 4).

**Figure 3:**
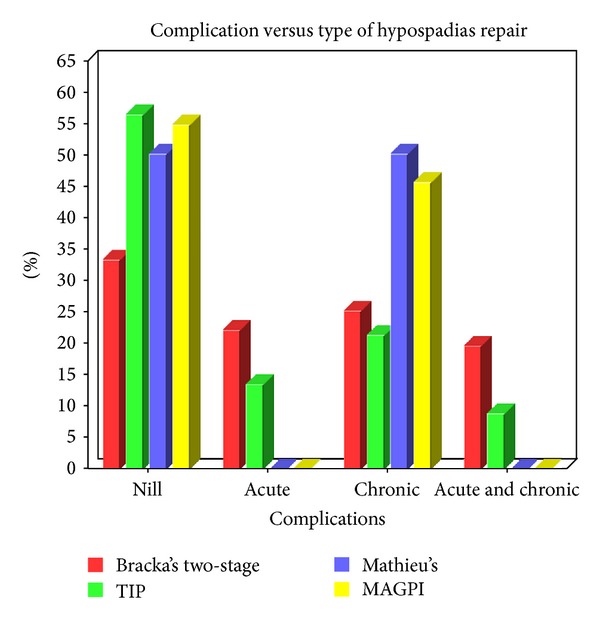
Complications versus type of hypospadias repair.

The frequencies of complications for two-stage hypospadias repair and single-stage repair were 66.9% (*n* = 218) and 44.1% (*n* = 45), respectively, which is statistically extremely significant with a *P* value of 0.0001, calculated by Fischer's exact test with a confidence interval of 95% (Table 5, Figure 3). The frequencies of UCF for two-stage repair and single-stage repair were 28.8% (*n* = 94) and 19.6% (*n* = 20), respectively, which is not statistically significant with *P*-value of 0.0728, calculated with Fischer's exact test with a confidence interval of 95% (Table 6).

## 4. Discussion

Hypospadias surgery is continuously evolving since its description by Celsius and Galen in the first and second centuries AD to improve suboptimal functional and cosmetic results. In spite of the achievements made in terms of establishing surgical protocols and improvements of short term results over the past 2 decades, the long term results are yet to be established. In the current study we evaluated the protocols, results, and effect modifiers of hypospadias repair at our centre.

In the current study only 1.87% and 34.6% patients were operated on in 1-2-year and 3–5-year age windows while the rest presented in ≥6 years of age. These are in contrary to the guidelines of the timing of hypospadias repair in the age of 6–18 months [4, 13]. The reason of low number of patients operated on in the 1st therapeutic window (6–18 months of age) is that we prefer hypospadias repair at preschool age (3-4 years) at our centre. The high ratio of patients presenting at age ≥6 years is due to lack of public awareness about the conditions and financial restraints.

Of the total study population 8.6% had positive family history for hypospadias. Akin et al. [19] observed positive family history in patients with hypospadias in 26.5% cases in their study in Turkey. Abdelrahman et al. [20] also observed 12% hypospadias patients with positive family history for this condition. Biased family history due to social stigma of hypospadias may be the reason behind this low frequency familial association in our study population.

Mid-penile hypospadias was the most common type followed by anterior hypospadias. Abdelrahman et al. [20] and Prat et al. [21] reported anterior hypospadias to be the most common type in their studies. In contrary to this Wu et al. [22] observed proximal hypospadias in 65.7% of their study population in China.

Chordee was associated with 74.3% of the hypospadias of the present study population and mild chordee was the most common type. Abdelrahman et al. [20] reported similar results with positive chordee in 88% cases.

Meatal abnormalities were associated with 9.6% cases of the hypospadias in the current study.

In contrast to 2.8% patients with hypospadias associated with cryptorchidism, Akin et al. [19] reported 14.7% patients of hypospadias with cryptorchidism. Abdelrahman et al. [20] observed this positive relationship between hypospadias and cryptorchidism in 20%. Wu et al. [22] also observed the same association in 7.3% cases.

In our study population 2.1% patients had inguinal hernia which is consistent with the results of Abdelrahman et al. [20] from Sudan who reported it in 2% of their study population. In contrast to our observations Wu WH et al. [22] observed inguinal hernia in 12.4% of hypospadias patients.

Two-stage (Bracka) repair was the most common procedure performed in 76.2% of cases. In 46.4% cases without chordee two-stage repair was performed for narrow urethral plate and incomplete glans cleft. Snodgrass' TIP was the second most common procedure performed in 20.8% cases. Abdelrahman et al. [20] performed MAGPI in most cases in their study population. Prat et al. [21] also performed single-stage procedure in most of their study population. The highest ratio of operators was the residents for two-stage repair (Bracka's). This overall high number of two-stage repairs is due to relative low expertise and awareness about single-stage procedures and relative short learning curve for two-stage repair.

The most common postoperative complication was edema observed in 28.3% cases. This high rate of postoperative edema may be due to prolonged tourniquet and inadvertent bipolar cautery use. UCF was noticed in 26.6% cases in the current study. Bhat and Mandal [23] observed UCF as the most common complication followed by edema in their PubMed literature review. Similar frequencies of UCF were reported by Chung et al. [24] in their study. Relatively lower frequency of 14.6% for UCF was observed by Huang et al. [25] in their study population. Bush et al. [26] reported UCF of 11.5% in their series of TIP repair. Snodgrass and Yucel [27] observed UCF of 33% and 10% for patients undergoing TIP with chromic catgut single layer closure and polyglactin subepithelial 2-layer closure, respectively. This relatively high frequency of UCF in our study population may be due to lack of routine use of magnification and microvascular instruments.

The overall frequency of complication and UCF was significantly higher for residents than specialist plastic surgeons which signify the long learning curve for hypospadias repair. Horowitz and Salzhauer [28] also reported results consistent with our observation about the learning curve of hypospadias repair.

The UCF frequency was higher for proximal hypospadias (penoscrotal, scrotal, and perineal) as compared to distal hypospadias which is consistent with the results of Chung et al. [24] study.

The overall frequency of complications was significantly high for two-stage repair than for single-stage repair. This may be because subjecting the patient to two surgeries 6 months apart prolongs psychological stress in the most vulnerable stage of life and cumulative donor site morbidity of the skin graft site. The two-stage repair of hypospadias also subjects patient to different operators at different level of expertise. There was no statistically significant difference noted in the frequency of UCF for single- and two-stage hypospadias repair.

To assess the long term outcome of hypospadias repair is a challenging job due to the difficulties in acquiring data. MacNeily et al. [29] reported excellent long term functional (voiding, sexual function, psychosexual adjustment, and self-appraisal) results despite initial higher frequencies of complications with a satisfaction rate of 86%.

To improve the outcome of hypospadias management we advocated the revision of existing institutional guidelines as repair of hypospadias in the initial window of 6–18 months, mandatory use of perioperative magnification, and increasing the threshold for two-stage Bracka's repair by revising its indications as severe degree of chordee, crippled cases with residual chordee, and fibrosed narrow urethral plate not correctable with Snodgraft repair. The expertise development in problem solving with single-stage repairs of hypospadias should be encouraged to minimize the psychological trauma and postoperative complications. In view of dedication required for management of hypospadias, the culture of casual hypospadias surgery should be abandoned and hypospadiology should be developed as separate surgical subspecialty.

## 5. Conclusion

Hypospadias surgery has a long learning curve because it requires a great deal of temperament, surgical skill, and acquaintance with magnifications along with knowledge of surgical anatomy. The most common type of hypospadias in the current study was distal penile and most of it was associated with chordee in our set-up. The most common complications were edema and UCF with a slightly higher prevalence than the ideal frequencies. The prevalence of postoperative complications can be reduced by establishing strict guidelines as operating within the recommended therapeutic windows, use of intraoperative magnifications and microsurgical instruments, strict direct supervision while residents are operating, and switching the preference and developing skills of single-stage repairs for more proximal hypospadias, hypospadias associated with mild to moderate chordee, and crippled cases. Regular periodic audit should also be performed to improve the outcome of the hypospadias repair.

## Figures and Tables

**Table 1:** Complications for different procedures.

Procedure	Complications				Total
	Nil	Acute complications	Chronic complications	Acute and chronic	
Two-stage repair	108 (33.1%)	72 (22.1%)	82 (25.1%)	64 (19.6%)	**326**
TIPS	50 (56.2%)	12 (13.5%)	19 (21.3%)	8 (8.9%)	**89**
Mathieu's repair	1 (50%)	0 (0%)	1 (50%)	0 (0%)	**2**
MAGPAI	6 (54.54%)	0 (0%)	5 (45.45%)	0 (0%)	**11**
Total	**165**	**84**	**107**	**72**	**428**

**Table 2:** Cross-tabulation of types of hypospadias and urethrocutaneous fistula.

Type of hypospadias	Urethrocutaneous fistula		Total
	Nil	Yes	
Glanular	25 (80.6%)	6 (19.4%)	**31**
Subcoronal	93 (74.4%)	32 (25.6%)	**125**
Distal penile	116 (75.8%)	37 (24.21%)	**153**
Midshaft	33 (70.2%)	14 (29.8%)	**47**
Proximal penile	6 (75%)	2 (25%)	**8**
Scrotal	1 (33.3%)	2 (66.7%)	**3**
Penoscrotal	10 (52.63%)	9 (47.34%)	**19**
Perineal	4 (66.7%)	2 (33.3%)	**6**
Crippled	26 (72.2%)	10 (27.8%)	**36**
Total	**314 (73.4%)**	**114 (26.6%)**	**428**

**Table 3:** Cross-tabulation of complications versus level of expertise.

Level of expertise	Complications		Total
	Yes	No	
Specialists	161 (56.9%)	122 (43.11%)	**283**
Residents	102 (70.34%)	43 (29.65%)	**145**
Total	**263**	**165**	**428**

*P* value = 0.0086 with a confidence interval of 95%, calculated by Fisher's exact test.

**Table 4:** Level of expertise versus corrected urethrocutaneous fistula cross-tabulation.

Level of expertise	Fistula		Total
	Nil	Yes	
Specialists	217 (76.7%)	66 (23.32%)	**283**
Residents	97 (66.9%)	48 (33.10%)	**145**
Total	**314 (73.4%)**	**114 (26.6%)**	**428**

*P* value = 0.0374, calculated by Fisher's exact test with a confidence interval of 95%.

**Table 5:** Type of surgical procedure versus complications cross-tabulation.

Type of surgical procedure	Complications		Total
	Yes	No	
Two-stage repair	218 (66.9%)	108 (33.1%)	**326**
Single-stage repair	45 (44.1%)	57 (55.9%)	**102**
Total	**165 (38.6%)**	**263 (61.4%)**	**428**

*P* value = 0.0001, calculated by Fisher's exact test with a confidence interval of 95%.

**Table 6:** Type of surgical procedure versus urethrocutaneous fistula cross-tabulation.

Type of surgical procedure	Fistula		Total
	Yes	No	
Two-stage repair	94 (28.8%)	232 (71.2%)	**326**
Single-stage repair	20 (19.6%)	82 (80.4%)	**102**
Total	**114 (26.6%)**	**314 (73.4%)**	**428**

*P* value = 0.0728, calculated by Fisher's exact test with a confidence interval of 95%.
